# In Situ Gel of Triamcinolone Acetonide-Loaded Solid Lipid Nanoparticles for Improved Topical Ocular Delivery: Tear Kinetics and Ocular Disposition Studies

**DOI:** 10.3390/nano9010033

**Published:** 2018-12-27

**Authors:** Akshaya Tatke, Narendar Dudhipala, Karthik Yadav Janga, Sai Prachetan Balguri, Bharathi Avula, Monica M. Jablonski, Soumyajit Majumdar

**Affiliations:** 1 Department of Pharmaceutics and Drug Delivery, School of Pharmacy, The University of Mississippi, MS 38677, USA; aatatke@go.olemiss.edu (A.T.); ndudhipa@olemiss.edu (N.D.); kjanga@go.olemiss.edu (K.Y.J.); prachetan2@gmail.com (S.P.B.); 2 Research Institute of Pharmaceutical Sciences, The University of Mississippi, MS 38677, USA; bavula@olemiss.edu; 3 National Center for Natural Products Research, The University of Mississippi, MS 38677, USA; 4 Department of Ophthalmology, Hamilton Eye Institute, University of Tennessee Health Science Center, Memphis, TN 38163, USA; mjablonski@uthsc.edu

**Keywords:** triamcinolone acetonide, solid lipid nanoparticles, in situ gel, optimal system, permeability, New Zealand rabbits, ocular distribution

## Abstract

Triamcinolone acetonide (TA), an intermediate acting corticosteroid, is used in the treatment of posterior ocular diseases, such as inflammation, posterior uveitis, and diabetic macular edema. The objective of this investigation was to prepare TA-loaded solid lipid nanoparticles (TA-SLNs) and in situ gel (TA-SLN-IG) formulations for delivery into the deeper ocular tissues through the topical route. TA-SLNs were prepared by hot homogenization and ultrasonication method using glyceryl monostearate and Compritol^®^ 888ATO as solid lipids and Tween^®^80 and Pluronic^®^ F-68 as surfactants. TA-SLNs were optimized and converted to TA-SLN-IG by the inclusion of gellan gum and evaluated for their rheological properties. In vitro transcorneal permeability and in vivo ocular distribution of the TA-SLNs and TA-SLN-IG were studied using isolated rabbit corneas and New Zealand albino rabbits, respectively, and compared with TA suspension, used as control (TA-C). Particle size, PDI, zeta potential, assay, and entrapment efficiency of TA-SLNs were in the range of 200–350 nm, 0.3–0.45, −52.31 to −64.35 mV, 70–98%, and 97–99%, respectively. TA-SLN-IG with 0.3% gellan gum exhibited better rheological properties. The transcorneal permeability of TA-SLN and TA-SLN-IG was 10.2 and 9.3-folds higher compared to TA-C. TA-SLN-IG showed maximum tear concentration at 2 h, indicating an improved pre-corneal residence time, as well as higher concentrations in aqueous humor, vitreous humor and cornea at 6 h, suggesting sustained delivery of the drug into the anterior and posterior segment ocular tissues, when compared to TA-SLN and TA-C. The results, therefore, demonstrate that the lipid based nanoparticulate system combined with the in situ gelling agents can be a promising drug delivery platform for the deeper ocular tissues.

## 1. Introduction

Ocular delivery of steroids has been extensively explored to treat various ophthalmic disorders including inflammation, uveitis, sympathetic ophthalmia, age-related macular degeneration (AMD), and retinal diseases [1]. Among various steroids, triamcinolone acetonide (TA), an intermediate-acting potent corticosteroid, chemically known as 9α-fluoro-11β,16α,17,21-tetrahydroxypregna-1,4-diene-3,20-dione cyclic 16,17-acetal with acetone, is frequently used in the management of ocular ailments [2].

Recently, TA intravitreal injections namely, Kenalog^®^, Kenacort^®^, Tricinolon^®^, and Flutex^®^, have gained substantial attention for the treatment of various ocular disorders, like temporal arteritis, uveitis, and inflammation, which are not responsive to other topical corticosteroids[3,4,5].Though this delivery gives a more targeted approach, it is associated with various complications, such as retinal hemorrhage or detachment, ocular hypertension, postoperative infectious, and non-infectious endophthalmitis, which might in turn lead to cataract formation or vision loss [6,7,8,9]. Further, TA is classified under BCS class IV, and thus shows both poor solubility and permeability. Because of this, formulating TA in the form of effective topical ophthalmic solutions or suspensions is a difficult task. 

Lipid based nanoparticulate systems have shown promising results in enhancing drug permeation across anatomical tissues, thus increasing overall efficacy [10,11]. Attempts have been made by various researchers to formulate TA topically in the form of aqueous nanomicellar drops [12], liposomes [13,14], and NLCs [4,5]. The results, however, in terms of drug loading and ocular tissue TA concentrations are either not promising or are lacking. Amongst different lipid nano carriers, solid lipid nanoparticles (SLNs) have also been used for effective drug delivery. SLNs are nano-sized lipid carriers, ranging from 50–1000 nm, having the capability to encapsulate lipophilic molecules inside the lipid matrix [15]. The drug is distributed into the lipid crystal lattice, thus increasing the overall stability of the formulation because of the presence of biodegradable lipid shell. SLNs are advantageous for lipophilic drugs that show poor solubility or permeability or both [16]. Previously, SLNs have been used as an ocular delivery platform to deliver various classes of drugs including anti-inflammatory [17], antibacterial [18], and anti-fungal [19] agents, showing benefits over other conventional formulation systems (solutions, suspension). However, TA-loaded SLNs (TA-SLNs) still remain an unexplored area in the field of topical ocular delivery.

Previously, Araújo et al., 2011 [5] developed the nano-structured lipid carriers (NLCs) of TA for the posterior segment of the eye. The ocular distribution/diffusion pathway of TA from the TA-NLC platform in vivo was, however, evaluated using confocal microscopy with the incorporation of Nile red in the NLC as the fluorescence marker. Thus, the study did not provide any insights into the TA levels in the deeper ocular tissues. In another study, Vallejo et al., 2018 [14] reported TA-loaded liposomes for topical delivery in rabbits and TA concentrations in the ocular tissues, post multiple dosing. Retina and vitreous humor was observed to reach peak TA concentrations at 12 h and declined subsequently.

*In situ* gelling systems are solutions at the time of administration but undergo sol-to-gel transformation [20] on contact with the physiological fluids or mucosa. Drug is released from the resulting gel in a sustained and controlled manner. The sol-to-gel transformation is triggered by the presence of electrolytes, pH, temperature, or UV light. The mechanism of gelation depends on the type of polymers used such as xanthan gum, hydroxyl propyl methyl cellulose, gellan gum, poloxamer, chitosan, etc. [21,22,23,24]. These *in situ* gelling systems, when instilled in the eye, form a gel by one of the above mechanisms and adheres to the corneal or conjunctival tissue, increasing the residence time of the formulation in the *cul-de-sac* and decreasing the dilution and loss of the formulation by tear outflow. In other words, the *in situ* gels overcome some of the major challenges faced by topical ophthalmic solutions or suspensions. 

The objective of the present investigation was to overcome the barriers encountered in topical ocular delivery by formulating TA-SLNs and further coupling it with an *in situ* gelling (TA-SLN-IG) system. The optimized TA-SLN and TA-SLN-IG formulations were evaluated for its in vivo ocular performance by studying TA tear kinetics and ocular distribution in New Zealand albino rabbits.

## 2. Materials and Methods

### 2.1. Materials

Compritol^®^ 888 ATO (glyceryl behenate) and glyceryl monostearate (GMS) were purchased from Gattefossé (Paramus, NJ, USA). TA, Pluronic^®^ F-68 (Poloxamer 188), Tween^®^ 80, gellan gum, amicon ultra centrifugal filter devices, high-performance liquid chromatography (HPLC)-grade solvents, liquid chromatography mass spectroscopy (LCMS) optima-grade solvents, and other analytical-grade chemicals were purchased from Fisher Scientific (Hampton, NH, USA). 

#### 2.1.1. Animal Tissues and Animals

Whole eyes of male albino New Zealand rabbits were ordered from Pel-Freez Biologicals (Rogers, AR, USA), and the tissues were used on the day of receipt. Male albino New Zealand rabbits were procured from Envigo (Indianapolis, IN, USA). All in vivo studies were performed as per the University of Mississippi Institutional Animal Care and Use Committee approved protocol (protocol number: 17-018).

### 2.2. Methods

#### 2.2.1. Preparation of TA-SLNs and TA-SLN-IG

TA-SLNs were prepared by using homogenization coupled with ultra-probe sonication method (i.e., film hydration method) [25] as per the composition given in Table 1. Briefly, the lipid phase, a combination of GMS and Compritol^®^ 888 ATO, was heated to 80 °C and TA was added under magnetic stirring until a clear drug-lipid phase was obtained. Simultaneously, the aqueous phase, consisting of Tween^®^ 80, Pluronic^®^ F-68, and glycerin (2.25%) in distilled water was heated, and transferred to the molten drug-lipid mixture under constant stirring, to form a pre-mixture. A pre-emulsion was obtained by emulsification process, using a T25 digital Ultra-Turrax (IKA, Wilmington, NC, USA) for 5 min, which was then subjected to probe sonication at 40% amplitude for 5 mins with 15 sec pulse on/off, using Sonics Vibra Cell Sonicator, Newtown, CT, USA)

TA-SLN-IG were prepared using the optimized TA-SLN formulation. The deionized water used in the preparation of SLNs was divided into two parts, one part to dissolve the gellan gum (0.2–0.6%) and the other for the aqueous phase as mentioned above. Both parts were heated to 80 °C and then transferred simultaneously to the hot lipid-drug mix, followed by emulsification and homogenization as carried out for TA-SLNs, to form in situ gels. The compositions of the various TA-SLN-IG formulations are presented in Table 1.

TA control (TA-C) suspension, 4% *w*/*v*, was prepared based on the composition information available for the marketed intravitreal injection Triesence^®^. The drug was suspended using sodium carboxy methyl cellulose (sodium CMC) as the suspending agent (Table 1), and a suitable volume was used in order to achieve the same concentration as the TA-SLN and TA-SLN-IG.

#### 2.2.2. Measurement of Particle Size, Zeta Potential, and Polydispersity Index

The particle size, zeta potential (ZP) and polydispersity index (PDI) of the TA-SLNs prepared were determined by photon correlation spectroscopy using a Zetasizer Nano ZS Zen3600 (Malvern Instruments, Westborough, MA, USA) at 25 °C in clear, disposable folded capillary cells. The particle size and PDI measurements were obtained using a helium-neon laser, and the data was analyzed based on the volume distribution. The samples were diluted 500 times with bi-distilled, 0.2 micron filtered water and measured for particle size and zeta potential in triplicate.

#### 2.2.3. Chromatographic Conditions for Sample Analysis

Drug content (assay), entrapment efficiency (EE) and in vitro permeability samples were analyzed using high performance liquid chromatography (HPLC) method. The HPLC system comprising of Waters 717 plus auto sampler, Waters 2487 dual absorbance detector, 600 Waters controller pump, and an Agilent 3395 Integrator. Luna^®^ C_18_ (4.6 mm × 250 mm) column, with a mobile phase of 1:1 isocratic solution of water and acetonitrile, at a flow rate of 1 mL/min and detection wavelength (λ_max_) of 254 nm, and AUFS of 1, was used for analysis of TA [26].

#### 2.2.4. Assay, Drug Loading, and Entrapment Efficiency

The drug content and loading in the TA-SLNs was determined by using the lipid precipitation method where, at first, the lipid in the SLN and SLN-IG (0.1 mL) was precipitated using 0.9 mL of methanol, followed by centrifugation (AccuSpin 17R centrifuge, Fisher Scientific, Hanover, IL, USA) under 13,000 rpm for 15 min and analyzing the supernatant using HPLC. 

Drug loading in SLNs were calculated using following formula:

Drug loading (%) = (Wt − Wu/W_L_) × 100 (1)

where Wt is the total weight of the drug, Wu is the weight of the unentrapped drug, and W_L_ is the weight of the lipids.

The EE(%) in TA-SLNs were calculated by estimating the concentration of the free drug in the aqueous phase of an undiluted formulation, using an ultrafiltration method with 100-kDa centrifugal filter unit (Amicon Ultra, Hanover, IL, Fisher Scientific, USA). Briefly, a 500 uL aliquot of the formulation was added to the filter unit and centrifuged under 13,000 rpm for 15 to 20 min. The filtrate was further diluted and analyzed for the drug content using HPLC-UV system. The EE was calculated by using the Equation (2):

(2) 00EE=[Di−DfDi]×   100

where D_i_ is the total drug content and D_f_ is the free drug present in the aqueous phase.

#### 2.2.5. Measurement of pH, Rheological, and *In Vitro* Gelling Characteristics of TA-SLN-IG

The pH of TA-SLN-IG formulations was measured using a calibrated pH meter (Mettler Toledo, Columbus, OH, USA). Simulated tear fluid (STF) (pH 7 ± 0.2), prepared by adding 0.678% sodium chloride, 0.0084% calcium chloride, 0.138% potassium chloride, and 0.218% sodium bicarbonate, in deionized water, was used as a medium for gelling the in situ agents. The gelling time is the time taken by the TA-SLN-IG formulations to form a gel when added to STF without any agitation. The gel residence time, considered as the time the formed gel remains intact in the STF, was also determined. The gelling time and gel residence time of the formulations with different concentration of gellan gum were determined by adding 50 µL of the TA-SLN-IG in 2 mL of freshly-prepared STF and maintaining the temperature of the glass vial at 34 °C in a water bath with continuous shaking. The reciprocation rate of the shaker was maintained at 100 time per minute. Viscosity of the formulations with or without the presence of STF was measured by a Brookfield cone and plate viscometer (LVDV-II+ Pro Viscometer, Middleboro, MA, USA). Gellan gum, with concentration ranging from 0.2% to 0.6%, was studied for in situ gelling properties. The amount of gel and STF used for studying the viscosity was 50 µL of the gel formulation with 7 µL of the STF [24]. 

#### 2.2.6. Stability Studies

The optimized TA-SLN (F5) formulation was subjected to moist-heat sterilization (121 °C for 60 min at 15 psi) in glass vials using a thermos-controlled autoclave (AMSCO Scientific Model SI-120, Columbia, MO, USA). The TA-SLN (F5) formulation was evaluated on the basis of the color, physical appearance, particle size, and compared before and after autoclaving.

Additionally, batches of TA-SLN (F5) and TA-SLN-IG (F13) were evaluated for their physical stability on storage up to four weeks at 40 °C/60% relative humidity (RH), 25 °C/75% RH and 4 °C. Samples were withdrawn at day 1 and day 28 and the particle size, PDI, drug content and EE of TA-SLN and rheological properties of TA-SLN-IG were determined, as per methods described in the sections above. 

#### 2.2.7. Differential Scanning Calorimetry (DSC)

A differential scanning calorimeter (DSC 25, TA instruments, New Castle, DE, USA) was employed to observe the melting and the recrystallization behavior of the drug with the excipients. The samples for the DSC analysis include TA and physical mixture of the lipid phase (with similar ratio as for formulation) melted and solidified. Approx. 5 mg of the samples, individually sealed in the aluminum pans, were placed over the sample platform. The reference pan, empty sealed aluminum pan, was placed on the reference platform. The pans were heated from 25 to 320 °C at the rate of 20 °C/min under nitrogen purge (20 mL/min).

#### 2.2.8. Fourier Transform Infrared Spectroscopy (FTIR)

The infrared spectra of the samples were obtained using Cary 660 series FTIR (Agilent Technologies, Santa Clara, CA, USA) and MIRacle ATR (attenuated total reflectance) systems. Pure drug, lipid excipients along with their physical mixtures and the final SLN formulation were studied for any interactions. 

#### 2.2.9. *In Vitro* Transcorneal Permeation Studies

Permeability studies were performed on the corneas isolated from rabbit whole eyes, acquired from Pel-Freez Biologicals (Rogers, AR, USA). The eyes were stored in Hanks’ balanced salt solution under ice-cold condition and shipped overnight. Immediately upon their receipt, the corneas were carefully separated, and used for the permeability studies. The isolated corneas were washed in ice-cold Dulbecco’s phosphate buffer saline (DPBS) solution, pH 7.4. The tissues were then mounted on Valia-Chien diffusion cells with the epithelial surface towards the donor chamber. The temperature of the diffusion cells was maintained at 34 °C with a circulating water bath, throughout the studies. 

TA concentration in TA-SLN (F5), TA-SLN-IG (F13), and TA-C were kept at 0.1% *w*/*v*, and about 1 mL of the formulation was added to the donor chamber of the respective diffusion cells. Three milliliters of DPBS with 5% *w*/*v* hydroxyl propyl beta cyclodextrin (HPβCD) solution was used as the receiver medium and stirred continuously with magnetic stirrer. Samples (600 µL) were withdrawn from the receiver chamber at the predetermined time points up to 2 h and replaced with an equal amount of DPBS-5% HPβCD solution to maintain sink conditions. The samples were stored at −80 °C until further analysis by HPLC. The analyses for all the samples were carried out in triplicate.

The cumulative amount of drug permeated (M_n_), steady state flux (J), and transcorneal permeability (P_eff_) across the rabbit cornea, were estimated in order to study the transport of TA across rabbit cornea [27]. The cumulative amount of TA was calculated as per the equation:

(3) $$M_{n}=\;V_{r}C_{r\left(n\right)}+\;\overset{x=n}{\underset{x=1}{\sum }}V_{s\left(x-1\right)}C_{r\left(x-1\right)}$$

where *n* is sampling time point; *V_r_* and *V_S_* are the volume in the receiver chamber (mL) and the volume of the sample collected at the nth time point (mL), respectively; and ***C_r(n)_*** is the concentration of the drug in the receiver chamber medium at nth time point (µg/mL).

The rate of TA transported across rabbit cornea was calculated using the slope of the cumulative amount of TA transported versus time plot. The steady state flux of TA was determined using the following equation:

(4) Flux(J)=(dM/dt)/A

where *M* is the cumulative amount of drug transported and *A* is the surface area of the cornea (0.636 cm^2^). 

The transcorneal permeability of TA was calculated by the following equation:

(5) $$Permeability\;\left(Papp\right)=\frac{\mathrm{Steady}\;\mathrm{state}\;\mathrm{flux}}{\mathrm{Donor}\;\mathrm{concentration}}$$

#### 2.2.10. Histology Studies

At the end of the in vitro permeation studies, the corneas that were exposed to medium (control), TA-SLN, TA-SLN-IG and TA-C formulations were used for histology studies. The isolated corneas were fixed with 4% paraformaldehyde for about 2 to 3 h. Later the corneas were rinsed with PBS, embedded in paraffin wax and sectioned at 5-micron thickness using a microtome (American Optical^®^ 820 Rotary Microtome, Labequip, Torrance, Ontario, Canada). The tissue sections were mounted on a slide and dried overnight in an oven. The slide was washed with xylene to remove the paraffin and the tissue was hydrated by washing with alcohol and water. The tissue was stained in nuclear dye Gill III hematoxylin (StatLab medical, Baltimore, MD, USA) by rinsing for 10 min and later counterstained with eosin. The sections were stained with hematoxylin and eosin, viewed on an Eclipse 800 photomicroscope (Nikon, Melville, NY, USA) and the images were captured using Picture Frame 3.0 software (Optronics), to record the morphological changes in the treated cornea, if any [28].

### 2.3. In Vivo Ocular Distribution Studies and Tear Kinetics

*In vivo* ocular disposition studies of TA-SLN (F5) and TA-SLN-IG (F13), in comparison with TA-C formulation, were carried out in New Zealand male albino rabbits, weighing between 2 to 3 kg, acquired from Envigo. The rabbits were allowed to acclimatize to the new surroundings, for one week, and the ocular disposition studies were then performed. Fifty microliters of the TA-SLN and TA-SLN-IG (0.1% *w*/*v*) formulations, and 40 µL of TA-C (4% *w*/*v*) was instilled into the *cul-de-sac* of the right eye while the left eye served as the control. About five microliters of tear was collected from the *cul-de-sac* of the test eye, using a micropipette, at every hour, up to 6 h. At the end of 6 h, the rabbits were euthanized with an overdose of pentobarbital administered via the marginal ear vein. The eye-globes were washed with ice cold DPBS and excised immediately. The ocular tissues were carefully isolated and were stored at −80 °C until further analysis. The tear samples were extracted in methanol and analyzed using the HPLC method described above in Section 2.2.3. The ocular tissue samples were extracted using methanol using the precipitation method and analyzed using UPLC-triple quadrupole (TQ)-MS system. All experiments were performed in triplicate.

#### 2.3.1. Sample Preparation

TA was extracted from the ocular tissues by protein precipitation technique. Briefly, the weights of the isolated ocular tissues: cornea, iris ciliary (IC), retina choroid (RC), and the sclera were noted, and the tissues were transferred to separate Eppendorf tubes. The tissues were further cut into small pieces and a suitable volume of 1 µg/mL prednisolone (PR) was added as the internal standard (IS) to each of the tissue sample and let stand. After that, 1 mL ice cold methanol with 0.1% formic acid was added as a protein precipitating solvent. Two hundred microliters of aqueous humor (AH) and 500 µL vitreous humor (VH) tissue proteins were precipitated using 200 µL and 500 µL of ice-cold methanol, respectively. To extract the drug into the solvent, all samples were vortexed for about 30 s, let stand at room temperature for 15 min and then sonicated for about 5 min. The samples were then centrifuged for 15 min at 13,000 rpm and the supernatant was collected and stored at –80 °C until further analysis. The samples were analyzed using the UPLC-triple quadrupole (TQ)-MS system.

#### 2.3.2. Bioanalytical Method

TA concentration in all the ocular tissues was quantified using an ultra performance liquid chromatography system coupled with a triple quadrupole mass spectrometer (UPLC-TQ-MS) (Waters, Milford, MA, USA). The peaks of both the drug and the IS (PR) were quantified with respect to the specific mass to charge (m/z) values (m/z 435 and 361 for TA and PR, respectively). Two microliters of the sample were eluted through a BEH C18 (100 mm × 2.1 m, 1.7 µm) Acquity UPLC^®^ column and separated using an isocratic mobile phase consisting of acetonitrile with 0.1% formic acid and water with 0.1% formic acid in the ratio of 98:2, respectively. The method showed a limit of detection (LOD) and quantification (LOQ) of 0.1 ng/mL for TA and IS, respectively. All the instrument functions were operated and managed by Mass Lynx software (version 4.1, Waters, Milford, MA, USA). 

#### 2.3.3. Statistical analysis

A one-way analysis of variance (ANOVA) along with Tukey’s post hoc test (version 5.00; GraphPad Prism Software, San Diego, CA, USA) was used to analyze the data obtained from the ocular distribution studies, and the statistically significant difference between the set of formulations was observed at a *p*-value less than 0.05 (*p* < 0.05).

## 3. Results and Discussion

### 3.1. Preparation and Physical Characterization of TA-SLNs

The main objective of the present work was to develop SLNs and their corresponding in situ gels for the sustained delivery of TA to the back of the eye and to determine the drug levels reaching the ocular tissues. The SLN formulation was designed, prepared and optimized using GMS, and Compritol^®^ 888 ATO as the lipid phase, selected based on the drug solubility in the lipid phase: Tween^®^ 80, Pluronic^®^ F-68, and glycerin constituted the aqueous phase. The percent content for all the excipient were below the limit in the inactive ingredients (IIG) database [29]. 

TA-SLNs were prepared using homogenization followed by probe sonication method. In order to develop and optimize the formulation, the influence of the lipid to surfactant ratio on the particle size and the PDI was studied. The composition of the formulation trials is given in the Table 1. It was observed that, when the surfactant ratio (Tween^®^ 80: Pluronic^®^ F-68) was kept constant at 1:1, the formulation with higher GMS content in the lipid ratio 1.7:0.3 (F1) showed higher particle size (400 ± 6.3 nm) and high PDI (0.47 ± 0.15). However, when the lipid content was decreased to 1:1, the particle size of the nanoparticles (F2) decreased (197.9 ± 5.2 nm), but with higher PDI (0.53 ± 0.23) compared with F1 formulation. This could be due to the amount and ratio of surfactant required to form the nanoparticles was enough, but was not sufficient to stabilize the nanoparticulate system.

On increasing the content of Tween^®^ 80 in the surfactant ratio from 1:1 to 9:1 (F3 and F4), there was a decrease in the particle size (280.7 ± 3.6 and 360.5 ± 4.3 nm) with the PDI still high (0.55 ± 0.16 and 0.492 ± 0.11), when compared to F1 (Table 2). This may be due to Pluronic^®^ F-68, which serves as the stabilizing agent between the nanoparticles and the aqueous phase—the reduction of which results in high PDI. Thus, the increase in Tween^®^ 80 content results in lower particle size, but a lower amount of Pluronic^®^ F-68 caused the PDI to increase.

Tween^®^ 80 content was decreased from 0.9 to 0.75 and Pluronic^®^ F-68 content was increased from 0.1 to 0.25, thus, maintaining a Tween^®^ 80 to Pluronic^®^ F-68 ratio of 3:1 (F5), and keeping the lipid ratio of 1.7:0.3. A decrease in both particle size (187.5 ± 1.8 nm) and PDI (0.35 ± 0.09) was observed, indicating that the amount of both surfactants was not only enough to emulsify the lipids with the aqueous phase and form solid lipid nanoparticles, but also to stabilize and disperse these nanoparticles throughout the aqueous phase [30]. 

It has been suggested that PDI values in the range of 0.01 to 0.5 represents a narrow enough distribution range. In the case of optimized TA-SLN (F5), the PDI value of 0.35 falls within this range and were, thus, considered to be as the more uniform formulation with high flux and high permeation as seen for in vitro release data [31].

ZP of TA-SLNs ranged from –32.2 ± 2.4 to –38.1 ± 2.1 mV, which is the indication of stability of the SLNs due to steric stabilization. In general, ZP of ± 30 mV is prerequisite for electrostatic stabilization of dispersed systems. Nevertheless, many experiments demonstrated that not only electrostatic repulsion, but also the steric stabilizer could impart stability to the SLN dispersion. In the case of TA-SLN, Pluronic^®^ F-68 was used in the formulation as surfactant. It is a non-ionic surfactant and reduced the electrostatic repulsion between the particles following steric stabilization of the nanoparticles by developing a coat around their surface for maintaining the stability of SLN [32].

The microscopic imaging studies of TA-SLNs were not conducted, but would correlate with the images reported with our previous reports [18]. Since the same SLN composition was used for the development of the TA nanocarriers.

### 3.2. Entrapment Efficiency, Drug Content, and Drug Loading of TA-SLNs

Another important factor to be considered in the optimization of SLNs is the amount of drug that is encapsulated in the nanoparticles and the drug content in the lipid matrix. On heating, the drug dissolves in the molten lipid matrix, and the amount of drug that gets encapsulated into the lipid matrix depends on the type of the lipids used [15]. The less ordered lipid matrix creates imperfections leading to void spaces in which drug molecules could be entrapped. From the results, the entrapment efficiency and total drug content in the lipid matrix was more than 90% (Table 2), which is due to higher solubility of the TA in the lipids, i.e., GMS and Compritol^®^ 888 ATO. Additionally, higher structural imperfections due to the crystalline nature of the lipids results in higher drug content entrapped in the lipid matrix. Drug loading of the TA-SLNs ranged from 3.68 ± 0.01 to 4.98 ± 0.01%. TA-SLNs were prepared with combination of mono and mixed triglycerides. Generally, Compritol SLN is expected to exhibit lower crystallinity and structural imperfections that create a space for drug molecules. Further, after cooling, Compritol^®^ recrystallizes in a series of polymorphs. Likewise, regarding the conditions employed during the preparation, TA could be homogenously dispersed in the lipid matrix, localized in a core, or deposited in the SLN shell. Formulation F5 showed lower particle size, low PDI with better ZP (−33 ± 2.5 mV), assay (95.43 ± 5.33), drug loading (4.98 ± 0.01%), and high EE (95.15 ± 1.3%) and, thus, was considered for developing and optimizing the TA-SLN-IG.

### 3.3. Preparation and Characterization of TA-SLN-IG

TA-SLN-IG formulations were developed by incorporating different concentrations of gellan gum into the optimized F5 formulation as per the Table 1 (F11–F15). Gellan gum is a polysaccharide that forms a clear gel in contact with the ions present in the tear film of the eye. Formation of the gel is due to presence of the mono or divalent cations that causes the cross-linking of the polymer. The introduction of the in situ gelling agent with SLN will retain the formulation in the *cul-de-sac* for a longer duration, thus, increasing the pre-corneal residence time [33]. Viscosity of the formulations in the presence or absence of STF, gelling time, and gel residence time were the parameters for the basis of selection of the concentration of the gellan gum, represented in Table 3.

For ophthalmic eye drops, viscosity of the formulation ranging from 25–50 cP are most widely considered, allowing for easy application [34]. The viscosity of the TA-SLN-IG was found to be in the range of 27.3 ± 3.2 to 220.2 ± 20.3 cP without STF and 105.6 ± 2.7 to > 3024 cP with STF. From the results, the viscosity of the formulation F13, (with concentration of 0.3% of gellan gum) was 43.8 ± 7.6 cP, which is in the range of ideal ophthalmic solution. Further, the viscosity of F13 with STF was found to be 531.9 ± 16.2 cP. The formulation will stay intact in the *cul-de-sac* following instillation (Table 3).

The viscosity of the optimized TA-SLN (F5) formulation (without in situ gel) was found to be 23.5 ± 3.5 cP. This in vitro data indicates the less viscous behavior of SLN compared with in situ gel post topical application.

Gel residence time was examined by comparing the total time the gel, formed on contact with STF, remains intact under constant agitation at 34 °C. The residence time for F11 and F13 is 2–3 and 6–7 h, respectively. Residence time for F14 was more than 10 h and for F12 and F15 was more than 24 h.

The pH of the TA-SLN-IG formulations was 6.8 ± 0.5, which is close to the lacrimal fluid pH. Therefore, TA-SLN--IG with 0.3% (F13) of gellan gum was considered as optimized and used for further studies.

### 3.4. Stability Studies

The SLN (F5) formulation was autoclaved at 121 °C for 60 min at 15 psi to examine the process stability of the product. Post sterilization, the physical characteristics of the formulation did not change and there was less than 5% change in the particle size and PDI. Similarly, the drug content and % EE was found to be in range (90.1 ± 3.8 and 92.8 ± 2.4, respectively) and was not statistically significant when compared to day 1 values (95.4 ± 5.4 and 97.5 ± 2.3, respectively). Hence, the study conveys that the formulation remains physically stable during the moist heat sterilization process. The four-week stability data of the F5 formulation kept at 4 °C, 25 °C, and 40 °C are presented in Table 4. The physical evaluation parameters, i.e., size, PDI, drug content, and EE, did not show any significant changes after 28 days compared to the results from day 1 (initial). This suggests that the TA-SLN colloidal dispersion formulations were stable for at least 28 days under the testing conditions. The rheological properties of the TA-SLN-IG (F13) formulation also remained unchanged up to four weeks—the last time point studied. Thus, based on the results, formulations F5 and F13 were considered as the optimized TA-SLN and TA-SLN-IG, respectively, and were further utilized in the in vitro permeation, tear kinetics, and ocular distribution studies.

### 3.5. Differential Scanning Calorimetry (DSC)

**Figure 1:**
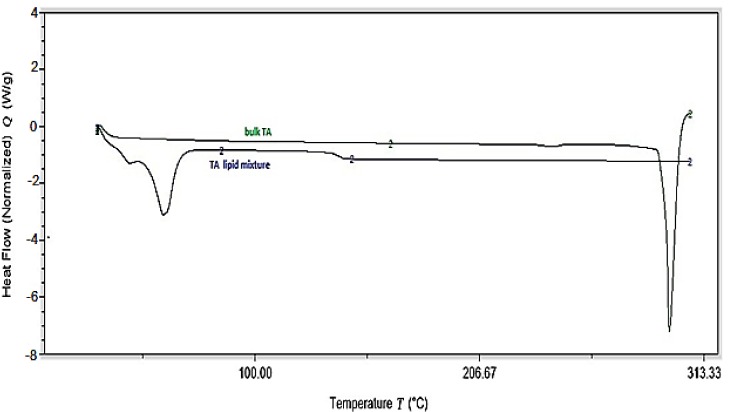
DSC thermograms of pure drug and physical mixture of drug-lipid.

Differential scanning calorimeter was used for analyzing the melting and recrystallization behavior of the drug within the lipid phase and is shown in Figure 1. The melting point of TA is reported as 293 °C (USP Monograph). A melting endothermic depression can be spotted at ~290 °C followed by the drug decomposition. The physical mixture of the drug in the lipid phase shows melting peak of the lipids at ~65 °C, coinciding with the melting point of Compritol^®^ 888 ATO. The drug-lipid mixture thermograms did not show any endothermic peak for TA, suggesting either complete solubility of the drug in the lipid phase or conversion of the drug from crystalline to amorphous form. Similar outcomes were also observed in earlier reported studies [25].

### 3.6. FTIR Studies

**Figure 2:**
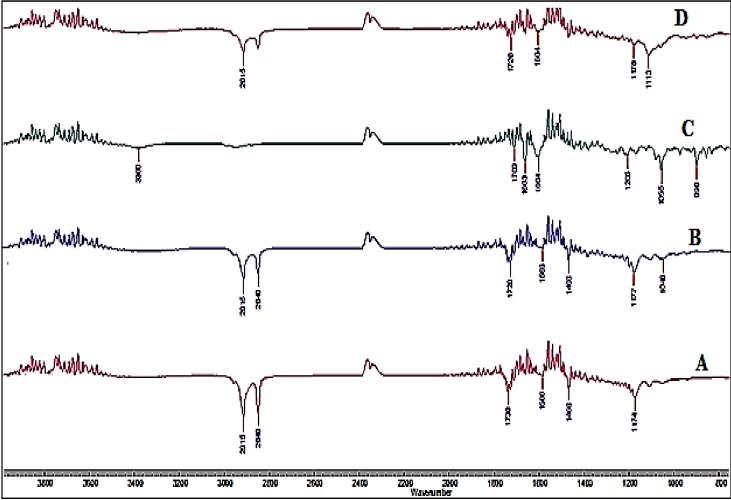
FTIR spectra of (**A**) Compritol^®^ 888 ATO, (**B**) glyceryl monosterate (GMS), (**C**) triamcinolone acetonide (TA), and (**D**) TA-SLN formulations.

FTIR spectrum was collected for pure TA, lipids i.e., Compritol^®^ 888 ATO, GMS, and the TA-SLN formulation to check for any interaction of the excipient with the drug during the formulation process. The FTIR spectras of TA, lipids and TA-SLN formulation are presented in Figure 2. For Compritol^®^ 888 ATO, GMS, and TA-SLN the major peak for % transmittance is observed at 2915 cm^−1^ which was assigned to C–H group. Another major peak for the bands from 1708–1738 cm^−1^, which corresponds to C=O group, and from 1586–1604 cm^–1^ corresponding to C=C group, were observed in all the excipient spectra. In the TA-SLN spectrum, peaks at similar band groups can be observed, which suggests that there was no interaction between the drug and lipid excipients. The variation in the peak intensities may be due to overlapping of certain functional groups for the specific band width.

### 3.7. In Vitro Transcorneal Permeation

Based on the saturated solubility studies, 5% *w*/*v* HPβCD in DPBS was used as the receiver medium for the transcorneal studies. About 5–7 microliters of STF was added to the donor compartment along with TA-SLN IG, to form a gel at the corneal surface, to mimic the in vivo conditions. Formulations TA-SLN (F5) and TA-SLN-IG (F13) were compared to TA-C for the in vitro permeation study.

**Figure 3:**
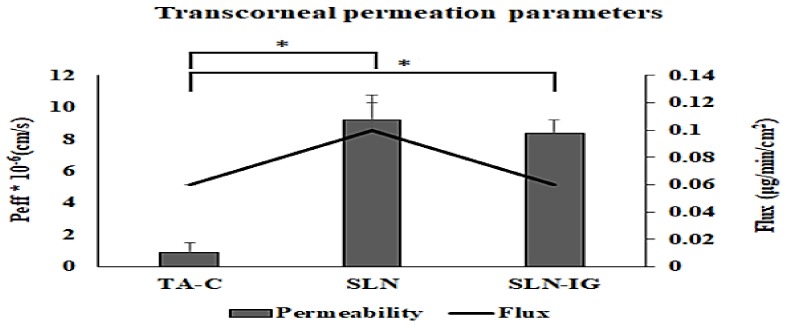
Transcorneal permeability profiles of TA from TA-SLNs (F5), TA-SLN-IG (F13), and TA-C through rabbit cornea (mean ± SD, n=3).

The permeability coefficient and flux for TA-C, TA-SLN (F5) and TA-SLN-IG (F13) is represented in Figure 3. Thetranscorneal permeability and flux of TA from TA-SLN (9.2 ± 1.65 × 10^−6^ cm/sec and 0.10 ± 0.02 μg/min/cm^2^) was significantly higher compared to that of the TA-C (0.89 ± 0.06 × 10^−6^ cm/sec and 0.057 ± 0.004 μg/min/cm^2^). This indicates that the lipid nanoparticles enhance the permeation of the drug through intact corneal tissues. The slightly lower flux (0.06 ± 0.00 μg/min/cm^2^) and permeability (8.4 ± 0.80 × 10^−6^ cm/sec) of TA from the TA-SLN-IG compared with TA-SLN indicates the controlled release of the drug from the higher viscosity formulation. These results were also observed in earlier reports [35].

### 3.8. Corneal Histology

**Figure 4:**
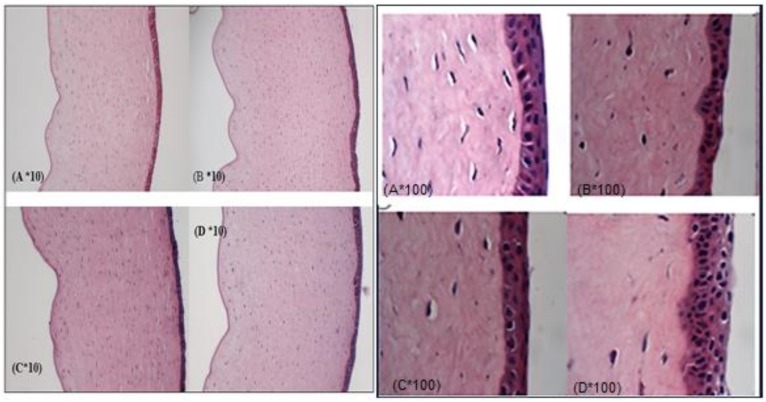
Corneal histology images of post trans-corneal permeation studies at 10× and 100× magnifications. Exposure of formulations: (**A**) TA-C, (**B**) TA-SLN, (**C**) TA-SLN-IG, and (**D**) DPBS.

Corneal histology studies were performed to examine the corneal tissue structure after the permeation studies were completed. Subsequently, after the 3 h transcorneal studies, corneas exposed to TA-SLN, TA-SLN-IG, and TA-C were utilized for histology studies and compared against corneas exposed to DPBS (control). The histological images for all four corneas are illustrated in Figure 4. The transverse section of cornea comprises of five layers, namely, epithelium, Bowman’s layer, stroma, Descemet’s layer, and endothelium. Any of the visual appearance like cut or rip in the layer, or missing layers or segregation of layers will be considered as toxic to the corneal tissues. However, the histology images of the dissected corneas show no such changes compared to the corneas treated with DPBS, thus suggesting that the formulations are not toxic to the ocular tissues [36,28].

### 3.9. In Vivo Animal Studies

#### 3.9.1. Tear Kinetics of TA-SLN and TA-SLN-IG

**Figure 5:**
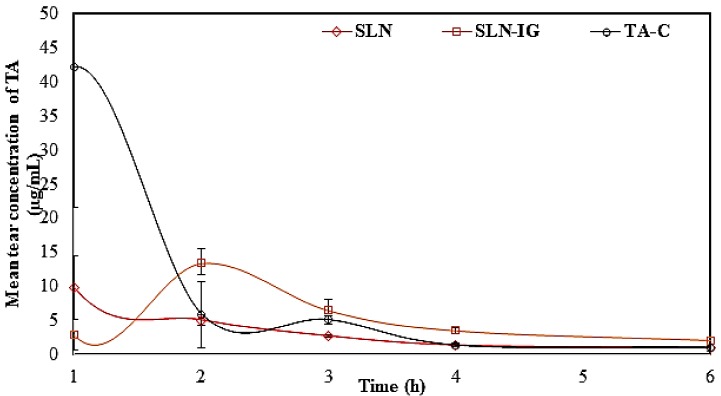
Mean tear concentration-time profiles of triamcinolone acetonide after topical administration of TA-SLN (F5), TA-SLN-IG (F13), and TA-C in New Zealand albino rabbits (mean ± SD, n = 3).

The tear kinetic studies of TA from TA-SLN, TA-SLN-IG, and TA-C were carried out in New Zealand albino rabbits and presented in Table 5. From the tear kinetic results, the highest concentration of the drug for the TA-C (4%) group was observed at first hour and which was approximately 42.2 μg/mL, with subsequent reduction in the drug concentration as a function of time, indicating low residence on the corneal surface (Figure 5). For TA-SLN (0.1%), highest drug concentration of approx. 10 μg/mL was observed at the first hour, whereas TA-SLN-IG (0.1%) showed maximum concentration (13.3 μg/mL) at the second hour. The mean residence time (MRT) and half-life (t_1/2_) of the TA from the TA-C, TA-SLN, and TA-SLN-IG formulation were found to be 1.2 ± 0.4, 2.0 ± 0.2, 2.7 ± 0.1 h and 1.7 ± 0.2, 3.7 ± 1.1, and 3.5 ± 0.4 h, respectively. AUC_total_ values were found to be 26.5 ± 8.0, 20.4 ± 2.0, and 29.9 ± 2.3 µg.h/mL, respectively, from TA-C, TA-SLN, and TA-SLN-IG. The greater value of t_1/2_ from SLN and SLN-IG compared to TA-C may be due to greater retention (entrapment in the mucin or mucoadhesion) as well as slower diffusion release of the drug from the lipid nanoparticles into tears followed by permeation through the ocular tissues. An advantage of the SLN formulations is that the nanoparticles are actively taken-up by the corneal and conjunctival epithelial cells. This is a feature of colloidal dispersions. As a result, compared to the TA-C formulation which contains micron sized TA particles, retention and uptake of the TA-SLNs are better than that of the simple suspension. The TA-SLNs are formulated using lipids (Compritol^®^ and GMS) and surfactants (Pluronic F68^®^ and Tween 80^®^). Moreover, due to the presence of lipid layers in the tear film, there is a possible interaction between SLNs and the tear film which in turn could lead to lipid depot formation and, thus, increase the mean residence time for SLN compared to TA-C in the conjunctival sac [10]. The presence of the gelling agents, SLN-IG, further improves the MRT [24]**.** Moreover, higher t_1/2_ and MRT of TA in tear observed with TA-SLN-IG explains the increased residence time of the formulation in the eye over the course of six hours. SLNs are supposed to be actively phagocytosed by the corneal and conjunctival cells. This is supposed to be a major advantage of colloidal dispersions. In addition to passive diffusion out of the SLNs, the lipases in the tear film and in the epithelial cells are responsible for the controlled release of TA. Release occurs both in the tear as well as in the corneal matrix [11,31,37]. Thus, tear kinetic study illustrates the sustained release model of the drug from lipid nanoparticles and in situ gel [38,39].

#### 3.9.2. Ocular Distribution Studies of TA-SLN and TA-SLN-IG

**Figure 6:**
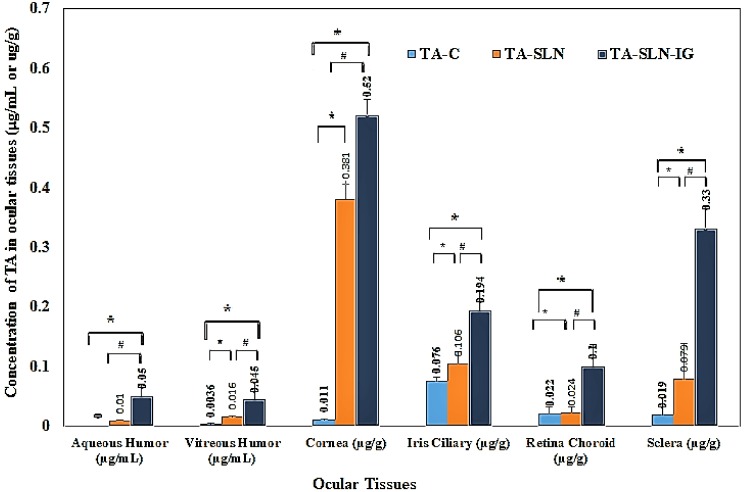
Concentration of TA in ocular tissues from TA-SLN (F5), TA-SLN-IG (F13), and TA-C at 6 h post dosing after topical administration in New Zealand albino rabbits (mean ± SD, n = 3).

The ocular distribution of TA in AH, VH, and the solid ocular tissues, 6 h post topical administration of TA-SLN, TA-SLN-IG, and TA-C formulations, are shown in Figure 6. Significantly higher concentrations of the drug were observed in AH with TA-SLN-IG formulation (0.05 ± 0.02 μg/mL) compared to TA-SLN (0.015 ± 0.004 μg/mL), With the TA-C formulation, TA levels were not detected 6 h post dosing. Similarly, significantly higher drug levels were observed from TA-SLN-IG (0.045 ± 0.028 μg/mL), TA-SLN formulation (0.016 ± 0.002 μg/mL) in VH compared to TA-C formulation (0.0036 ± 0.0009 μg/mL). 

The TA concentrations in cornea and IC were found to be 0.011 ± 0.001 and 0.076 ± 0.006 μg/g for TA-C, 0.381 ± 0.024 and 0.106 ± 0.019 μg/g TA-SLN, and 0.52 ± 0.027 and 0.194 ± 0.033 μg/g for TA-SLN-IG, respectively. Concentrations of TA in RC and sclera were 0.022 ± 0.01 μg/g, 0.019 ± 0.03 μg/g and 0.024 ± 0.009 μg/g, 0.079 ± 0.059 μg/g and 0.1 ± 0.037 μg/g, 0.33 ± 0.038 μg/g, for TA-C, TA-SLN, and TA-SLN-IG formulation, respectively. For both cornea and IC, the drug levels obtained from the TA-SLN-IG formulation was determined to be significantly higher than that from TA-SLN.

LN and TA-C formulations. Similarly, for the posterior segment tissues, i.e., sclera and RC, the drug concentration from TA-SLN-IG were significantly higher. The drug concentration in cornea, IC, RC, and sclera were approx. 47.2-, 2.5-, 4.5-, and 17.3-folds higher compared to TA-C and 1.3-, 1.8-, 4.1-, and 4.1-folds compared to concentration by TA-SLN formulation. 

Thus, higher drug levels are observed in the ocular tissue on administration of TA-SLN-IG owing to enhanced residence time of the TA-SLN-IG on the ocular surface due to cross-linking of the polymer chains mediated by the cations in the tear fluid, leading to gel formation on the ocular surface, which contributes to an extended corneal contact time [40]. These findings are consistent with earlier reports [41,42].

Previously, Araújo et al., 2011 [5] developed and reported TA-NLC formulations for ocular delivery. The performance of the TA-NLCs after topical application into the mice eyes was gauged using fluorescence intensity measurement, using confocal microscope, of Nile red (NR), which was used as a fluorescent lipid marker. From the results, strong fluorescence was observed on the surface of the anterior segment for TA-NLC treated eyes in the first 8 min and was detectable at 160 min also. However, fluorescence was not detected in the case of eyes treated with NR solution, either through the systemic, corneal or non-corneal pathways in the 160 min experiment duration. The study, however, did not present any data on the TA concentrations achieved in the ocular tissues. Moreover, compared with the TA-NLC reported by Araújo et al., 2011, drug loading of TA was increased from 0.025 to 0.1% in the TA-SLN and TA-SLN-IG formulations of the present study, a four-fold increase.

In another study, Altamirano-Vallejo et al. [14] developed topical TA liposomes for vitreoretinal delivery. Four groups with five rabbits each, were treated with topical TA liposome formulation every 2 h, six times, during 14 days, and the drug concentration was evaluated at the end of 12 h, 1, 7, and 14 days. The highest levels of TA were observed at the end of 12 h in retina and VH (0.252 µg/g and 0.036 µg/g, respectively), and eventually decreased at the end of the 14^th^ day following a first-order elimination kinetics.

In this study, high levels of TA were observed in ocular tissues from TA-SLN-IG with about 0.045 µg/mL TA in VH at the end of 6 h, with a single dosing administered topically. Further, TA-SLN and TA-SLN-IG showed peak concentrations after 1 h and 2 h, respectively, in tear suggesting an increased retention time of the formulation in the *cul-de-sac*. Therefore, TA-SLN-IG ophthalmic formulations could be an alternative approach for topical delivery of TA.

## 4. Conclusion

TA-loaded SLNs and the in situ gel formulations were successfully prepared and optimized. The transcorneal permeability studies showed improved permeability when compared to the control suspension. The histology of the treated corneas indicated no damage to the corneal epithelial tissues. TA-SLN-IG generated higher drug concentration in tear and in the anterior and posterior segment ocular tissues compared to TA-SLN and the control suspension. Overall, SLN and SLN-IG studied in this work appear to be suitable for ocular absorption enhancement of TA possibly acting via multidimensional mechanisms, namely, by prolonged drug residence time in the ocular surface and conjunctival sac, by sustained drug release from the delivery system, and/or by reduced pre-corneal drug loss, compared with various novel drug delivery systems. Thus, the SLNs combined with in situ gelling agents demonstrates an efficient topical drug delivery platform.

## Figures and Tables

**Table 1:** Composition of triamcinolone acetonide loaded solid lipid nanoparticles and in situ gels.

Formulation Composition (%)	F1	F2	F3	F4	F5 *	F11	F12	F13 ^†^	F14	F15	TA-C
**Triamcinolone acetonide**	0.1	0.1	0.1	0.1	0.1	0.1	0.1	0.1	0.1	0.1	4
**Glyceryl mono stearate (GMS)**	1.7	1	1.7	1.5	1.7	1.7	1.7	1.7	1.7	1.7	-
**Compritol^®^ 888 ATO**	0.3	1	0.3	0.5	0.3	0.3	0.3	0.3	0.3	0.3	-
**Tween ^®^ 80**	0.5	0.5	0.9	0.9	0.75	0.75	0.75	0.75	0.75	0.75	-
**Pluronic^®^ F-68**	0.5	0.5	0.1	0.1	0.25	0.25	0.25	0.25	0.25	0.25	-
**Glycerin**	2.25	2.25	2.25	2.25	2.25	2.25	2.25	2.25	2.25	2.25	-
**Gellan gum**	--	--	--	--	--	0.2	0.5	0.3	0.4	0.6	-
**Sodium Carboxy** **Methyl Cellulose**	--	--	--	--	--	--	--	--	--	--	0.5
**Water**	100	100	100	100	100	100	100	100	100	100	100

* and ^†^ indicate the optimized TA-SLN formulation and SLN-IG formulation, respectively. Each ingredient in composition is expressed in %*w*/*v*. TA-C indicate control suspension.

**Table 2:** Physical characteristics – size, PDI, ZP, assay, drug loading, entrapment efficiency and viscosity of triamcinolone acetonide loaded solid lipid nanoparticles (mean ± SD, n = 3).

Formulation	Size (nm)	PDI	ZP (mV)	Entrapment Efficiency (%)	Assay (%)	Drug Loading (%)	Viscosity (cP)
**F1**	400 ± 6.3	0.47 ± 0.15	−32.2 ± 2.4	91.4 ± 3.64	92.31 ± 1.31	4.96 ± 0.01	24.15 ± 2.6
**F2**	197.9 ± 5.2	0.53 ± 0.23	−38.1 ± 2.1	93.4 ± 2.1	81.45 ± 4.60	3.68 ± 0.01	22.62 ± 9.2
**F3**	280.7 ± 3.6	0.55 ± 0.16	−36.3 ± 1.9	90.0 ± 2.96	90.42 ± 5.70	4.95 ± 0.01	20.43 ± 2.7
**F4**	360.5 ± 4.3	0.49 ± 0.11	−35 ± 3.3	92.8 ± 2.37	85.2 ± 4.20	4.96 ± 0.01	22.91 ± 2.1
**F5**	187.5 ± 1.8	0.35 ± 0.09	−33 ± 2.5	95.1 ± 1.3	95.43 ± 5.33	4.98 ± 0.01	23.51 ± 3.5

**Table 3:** Rheological properties of triamcinolone acetonide loaded solid lipid nanoparticle in situ gels (mean ± SD, n = 3).

Formulation	Gellan Gum (%)	*In vitro* Gelling Time	Gel Residence Time (h)	Viscosity (cP) Without STF	Viscosity (cP) With STF
**F11**	0.2	Immediate	2–3	27.32 ± 3.2	105.6 ± 2.7
**F13**	0.3	Immediate	6–7	43.8 ± 7.6	531.9 ± 6.2
**F 14**	0.4	Immediate	> 10	70.8 ± 15.5	1296.7 ± 8.9
**F 12**	0.5	Immediate	> 24	185.2 ± 20.3	2246.8 ± 5.4
**F 15**	0.6	Immediate	> 24	220.2 ± 20.3	> 3024

**Table 4:** Stability studies of optimized TA-SLN formulation (F5) at different temperatures for one month (mean ± SD, n = 3).

Duration	Condition	Size (nm)	PDI	Assay (%)	Entrapment Efficiency (%)
**Day 1**	-	187.5 ± 1.8	0.35 ± 0.09	95.42 ± 5.4	97.56 ± 2.3
	4 °C	188.3 ± 4.1	0.32 ± 0.11	92.94 ± 4.8	94.52 ± 3.3
**Week 4**	25 °C	195.6 ±15.6	0.31 ± 0.13	91.65 ± 6.7	96.02 ± 1.8
	40 °C	200.3 ± 9.6	0.33 ± 0.21	89.55 ± 4.4	93.68 ± 2.7

**Table 5:** Ocular tear pharmacokinetic parameters of triamcinolone acetonide at the end of 6 h after the topical administration of TA-SLN (F5), TA-SLN-IG (F13), and TA-C in New Zealand albino rabbits (mean ± SD, n = 3).

Pharmacokinetic Parameters	TA-C	TA-SLN	TA-SLN-IG
**C_max_ (µg/mL)**	42.2	9.75	13.38 ^#^
**t_max_ (h)**	1	1	2 ^#^
**AUC_0-6_ (µg·h/mL)**	26.52 ± 8.04	20.41 ± 2.07	29.99 ± 2.39 ^#^
**MRT (h)**	1.29 ± 0.42	2.04 ± 0.20 ^*^	2.75 ± 0.11 ^*^
**t_1/2_ (h)**	1.72 ± 0.29	3.76 ± 1.14 ^*^	3.54 ± 0.42 ^*^

^*^ indicates *p* < 0.05 when TA-SLN/TA-SLN-IG are compared with TA-C. ^#^ indicates *p* < 0.05 when TA-SLN-IG compared with TA-SLN.
